# Growth factor–induced activation of MSK2 leads to phosphorylation of H3K9me2S10 and corresponding changes in gene expression

**DOI:** 10.1126/sciadv.adm9518

**Published:** 2024-03-13

**Authors:** Karen G. Wong, Yu-Chia F. Cheng, Vincent H. Wu, Anna A. Kiseleva, Jun Li, Andrey Poleshko, Cheryl L. Smith, Jonathan A. Epstein

**Affiliations:** ^1^ Department of Cell and Developmental Biology, Penn Epigenetics Institute, Perelman School of Medicine, University of Pennsylvania, Philadelphia, PA 19104, USA.; ^2^ Department of Microbiology, Perelman School of Medicine, University of Pennsylvania, Philadelphia, PA 19104, USA.; ^3^ Department of Medicine and Penn Cardiovascular Institute, Perelman School of Medicine, University of Pennsylvania, Philadelphia, PA 19104, USA.

## Abstract

Extracellular signals are transmitted through kinase cascades to modulate gene expression, but it remains unclear how epigenetic changes regulate this response. Here, we provide evidence that growth factor–stimulated changes in the transcript levels of many responsive genes are accompanied by increases in histone phosphorylation levels, specifically at histone H3 serine-10 when the adjacent lysine-9 is dimethylated (H3K9me2S10). Imaging and proteomic approaches show that epidermal growth factor (EGF) stimulation results in H3K9me2S10 phosphorylation, which occurs in genomic regions enriched for regulatory enhancers of EGF-responsive genes. We also demonstrate that the EGF-induced increase in H3K9me2S10ph is dependent on the nuclear kinase MSK2, and this subset of EGF-induced genes is dependent on MSK2 for transcription. Together, our work indicates that growth factor–induced changes in chromatin state can mediate the activation of downstream genes.

## Teaser

MSK2 kinase is activated by growth factor signaling to phosphorylate histones at enhancers of growth factor–responsive genes.

## INTRODUCTION

A coordinated response to external stimuli is important for numerous cellular processes and the overall health of cells and organisms. Common extracellular signals such as growth factors, temperature changes, and environmental stress trigger signaling cascades that alter gene expression and modify cellular function. While the transcriptional effects of growth factor stimulation are well established, the impacts of signaling on chromatin organization that in turn directs changes in gene expression are not as well understood. Extracellular signaling can modulate transcription factors, but signaling has also been shown to lead to changes in histone modifications, which then promote variations in gene expression. For example, treating quiescent cells with various mitogens and growth factors results in rapid histone serine phosphorylation and transcriptional activation (*1*, *2*), and fluctuations in cellular metabolites modulate the activity of many DNA- and chromatin-modifying enzymes that use these metabolites as substrates or cofactors (*3*–*5*). Thus, the proper balance of and reception to external stimuli can have direct effects on regulating epigenetic modifications, and the mechanisms by which changes in chromatin organization result from extracellular signaling warrant further study.

Chromatin organization within the nucleus affects transcriptional regulation and is important for maintaining cellular function and identity (*6*–*10*). Posttranslational histone modifications are associated with the state of various genomic elements such as promoters, enhancers, or actively transcribed genes. Additionally, histone modifications are recognized by different chromatin-binding proteins that can recruit secondary enzymes and complexes to a particular site in the genome, enabling the cell to translate these modifications into changes in chromatin accessibility, transcriptional activity, or nuclear localization (*11*).

While chromatin-interacting proteins recognize specific histone modifications, a functional epitope within the histone tail may be driven by more than just a single modification. For example, phosphorylation of the serine-10 residue on histone H3 (H3S10) can diminish the binding affinity between HP1 and H3K9me3 both through steric hindrance and by disrupting hydrogen bonds in the binding pocket that stabilize the interaction (*12*–*14*). H3S10 phosphorylation has been shown to evict HP1 and other reader proteins from chromatin, allowing for chromatin condensation and mitosis to proceed (*15*). This dynamic shift in protein-chromatin interaction in response to the modification status of adjacent histone tail residues has been termed the “phospho-methyl switch” (*16*) and has been demonstrated in several other contexts. Phosphorylation of H3T3 reduces the binding of TFIID subunits to H3K4me3 and prevents transcription initiation (*17*), while the serotonylation of H3Q5 has been shown to enhance this binding (*18*). Further, the phosphorylation of H3T6 by PRKCβ prevents LSD1 from recognizing H3K4me1/2 as a target for demethylation during androgen receptor signaling, thereby prolonging androgen-dependent gene activation (*19*). Thus, the residues surrounding a histone modification are also crucial to epitope recognition and binding.

The histone modification H3K9me2 marks 30 to 50% of the genome, making it one of the most prevalent histone modifications in the cell (*20*). H3K9me2 is mainly associated with transcriptionally repressed, densely packed heterochromatin and forms domains of hundreds of kilobases, many of which overlap with lamina-associated domains (LADs) and are positioned at the nuclear periphery (*7*, *21*–*24*). Lamina-chromatin interactions have been widely demonstrated to regulate gene expression changes and lineage specification (*7*, *8*, *25*). Previous work has also demonstrated that a small subset of H3K9me2 domains do not interact tightly with lamina proteins [H3K9me2-only domains (KODs)] and are specifically enriched for enhancers (*26*). While regions of H3K9me2 are largely the same across different cell types, reduction of H3K9me2 at some lineage-specific genes and/or their regulatory elements has been shown to correlate with differentiation and changes in gene expression (*7*, *20*, *25*). However, the molecular mechanisms that mediate H3K9me2 levels, changes in nuclear localization, or increased accessibility to enable transcription of these regions are poorly understood.

Previous work from our group and others has shown that an H3K9me2/S10ph phospho-methyl switch facilitates large-scale detachment of H3K9me2-marked chromatin from the nuclear lamina at the onset of mitosis (*24*, *27*). Here, we describe an H3K9me2/S10ph switch that occurs during interphase in response to growth factor signaling. We show that epidermal growth factor (EGF) stimulation results in an increase in the cellular abundance of H3K9me2S10ph, and we identify MSK2 [ribosomal protein S6 kinase A4 (*RPS6KA4*)] as a nuclear kinase that mediates this phosphorylation. We describe a phosphorylation substrate, H3K9me2S10, that has not to our knowledge been previously linked to growth factor signaling in human cells, and we demonstrate the relevance of the combinatorial histone modification H3K9me2S10ph in transcriptional activation in response to EGF stimulation. Together, our results suggest a mechanism through which extracellular signals induce changes in the epigenetic landscape that ultimately affect gene expression and the cellular response to stimuli.

## RESULTS

### EGF stimulation leads to an increase in the cellular abundance of H3K9me2S10ph

**Fig. 1.**
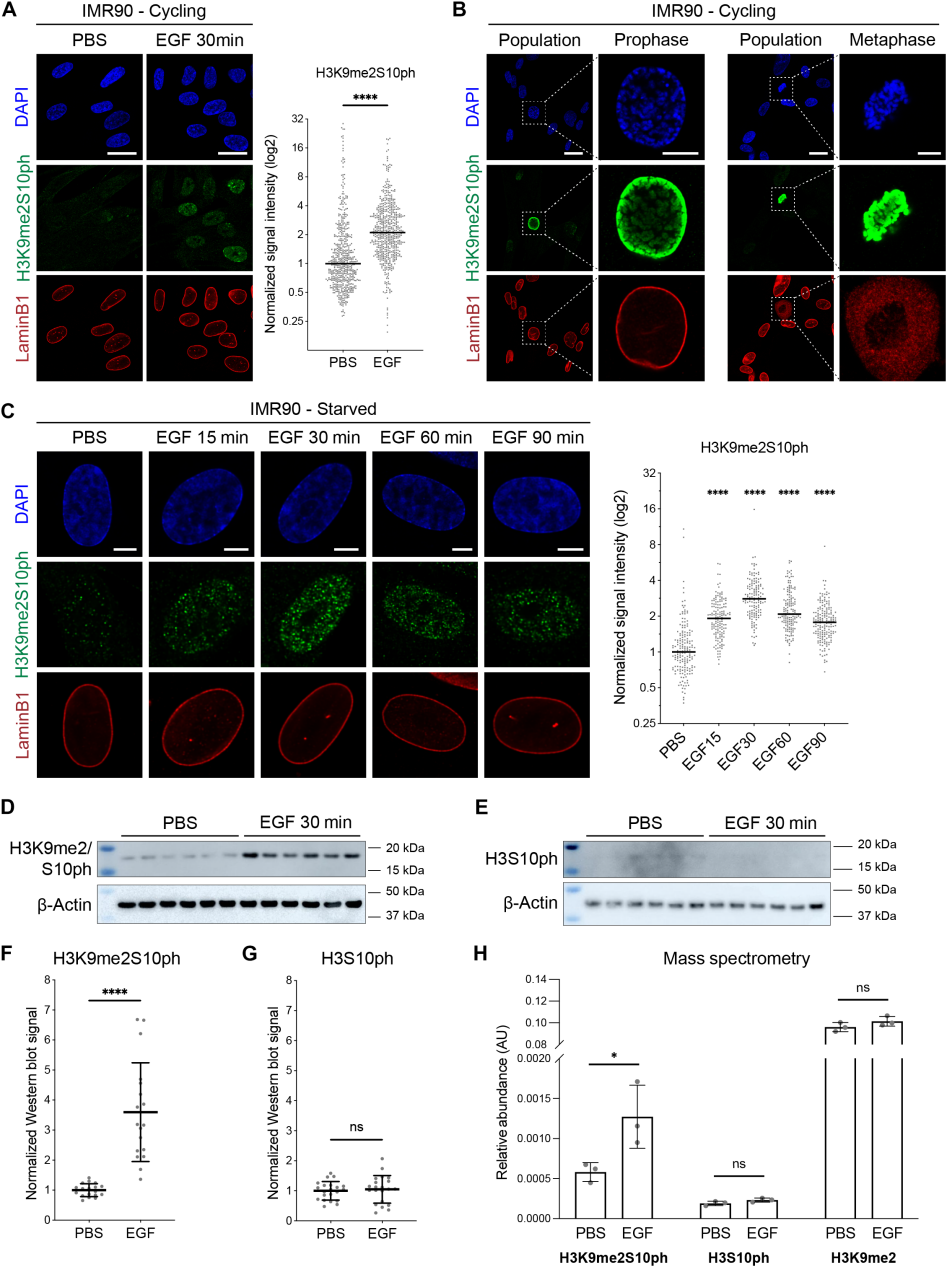
EGF stimulation leads to an increase in the cellular abundance of H3K9me2S10ph. (**A**) Representative confocal images of cycling IMR90 cells immunostained for H3K9me2S10ph (green) and lamin B1 (red) and counterstained with DAPI (blue). Cells were treated with PBS (vehicle control) or EGF for 30 min. Scale bars, 25 μm. Dot plot shows normalized H3K9me2S10ph signal intensities. Lines show median values. *n* ≥ 564 cells per condition. (**B**) Representative confocal images of cycling IMR90 cells immunostained as in (A). Inset shows an individual nucleus of a cell in prophase (left) or metaphase (right). Scale bars, 25 and 5 μm. (**C**) Representative confocal images of serum-starved IMR90 cells treated with EGF for the indicated times; staining and quantification as in (A). *n* ≥ 140 cells per condition. Scale bars, 5 μm. (**D** and **E**) Representative Western blots of IMR90 cells treated with PBS or EGF for 30 min and probed for (D) H3K9me2S10ph or (E) H3S10ph and loading control (β-actin). (**F** and **G**) Quantifications of Western blots shown in (D) and (E). H3K9me2S10ph and H3S10ph signals, normalized to loading control (β-actin), and the mean of the signal in the PBS samples. Lines indicate mean ± SD. *n* = 18 (D) and 21 (E) biological replicates per condition. (**H**) Quantification using mass spectrometry of posttranslational modifications of the H3 tail from histones isolated from IMR90 cells treated with PBS or EGF for 30 min. *n* = 3 biological replicates. Statistical analyses performed using unpaired *t* test [(A), (F), (G), and (H)] or two-way analysis of variance (ANOVA) (C). ****P < 0.0001; *P < 0.05; ns, not significant.

To determine the effect of EGF stimulation on cellular levels of H3K9me2S10ph, we treated human lung fibroblast IMR90 cells with recombinant human EGF for 30 min and performed immunofluorescence (IF) staining for H3K9me2S10ph. We observed a significant increase of H3K9me2S10ph in EGF-treated cells compared to untreated [phosphate-buffered saline (PBS)] cells as quantified by signal intensity within the nucleus (Fig. 1A). This effect was not limited to IMR90 cells. A significant increase of H3K9me2S10ph was also induced by EGF in a distinct cell line, RPE1 (human retinal pigment epithelial) cells (fig. S1A).

In both PBS- and EGF-treated samples, we observed a small population of cells with high H3K9me2S10ph that displayed characteristics of cells in mitosis (Fig. 1B). This is consistent with our previous work describing elevated H3K9me2S10ph levels in mitotic cells compared to interphase cells (*24*). To identify those cells in late G_2_-M phase, we performed IF for Aurora B kinase (AKB) in cycling cells. We excluded the AKB-positive cells and quantified H3K9me2S10ph signal in the remaining interphase cells with or without EGF treatment and recapitulated our result that EGF treatment leads to an increase in H3K9me2S10ph in interphase cells (fig. S1B). To focus our study on the effect of EGF on H3K9me2S10ph in interphase, we arrested cells in G_0_-G_1_ by serum starvation. Cells that were serum-starved for 24 hours and then treated with EGF for 30 min also showed a significant increase of H3K9me2S10ph by IF in both IMR90 (Fig. 1C) and RPE1 cells (fig. S1C). EGF stimulation of H3K9me2S10ph peaked at 30 min, after which there was a steady decrease (Fig. 1C). We note that this is not a result of EGF inducing cells to enter mitosis as EGF stimulation did not change the cell cycle profile of the cells (fig. S1D). Another growth factor, platelet-derived growth factor (PDGF), induced a similar significant increase in H3K9me2S10ph in serum-starved RPE1 cells (fig. S1E).

We confirmed EGF-induced changes in H3K9me2S10ph levels by Western blot analysis of serum-starved IMR90 cells following 30 min of EGF treatment. Consistent with the imaging experiments, we observed a significant increase in H3K9me2S10ph in the EGF-treated samples compared to untreated cells (Fig. 1, D and F). Since numerous studies have identified H3S10 as a common substrate for phosphorylation (*28*), we also examined H3S10ph levels before and after 30 min of EGF stimulation. We did not detect an increase of H3S10ph in response to EGF in that time frame (Fig. 1, E and G).

We validated the antibodies used to detect histone posttranslational modifications by probing peptides containing combinations of relevant H3 tail modifications. Each antibody was specific for only the given modification and displayed no cross-reactivity (fig. S1F). Specifically, the anti-H3K9me2S10ph antibody did not recognize H3S10ph; nor did the H3S10ph antibody bind to the H3K9me2S10ph peptide. This is consistent with previous antibody validation using a commercial histone peptide array (*7*, *24*).

To further validate our findings using an antibody-independent method, we performed mass spectrometry on acid-extracted histones from serum-starved IMR90 cells treated for 30 min with EGF and again detected higher levels of H3K9me2S10ph in the EGF-treated samples compared to untreated cells (Fig. 1H). In agreement with the Western blot results, mass spectrometry analysis showed no significant increase in H3S10ph in response to EGF, and total H3K9me2 levels also did not change (Fig. 1H). On the basis of IF, Western blot, and mass spectrometry, we conclude that EGF stimulation of interphase IMR90 cells induces phosphorylation of H3S10 when the adjacent lysine-9 is dimethylated, but not when lysine-9 is unmodified.

### EGF induction of H3K9me2S10ph is MSK2 dependent

**Fig. 2.**
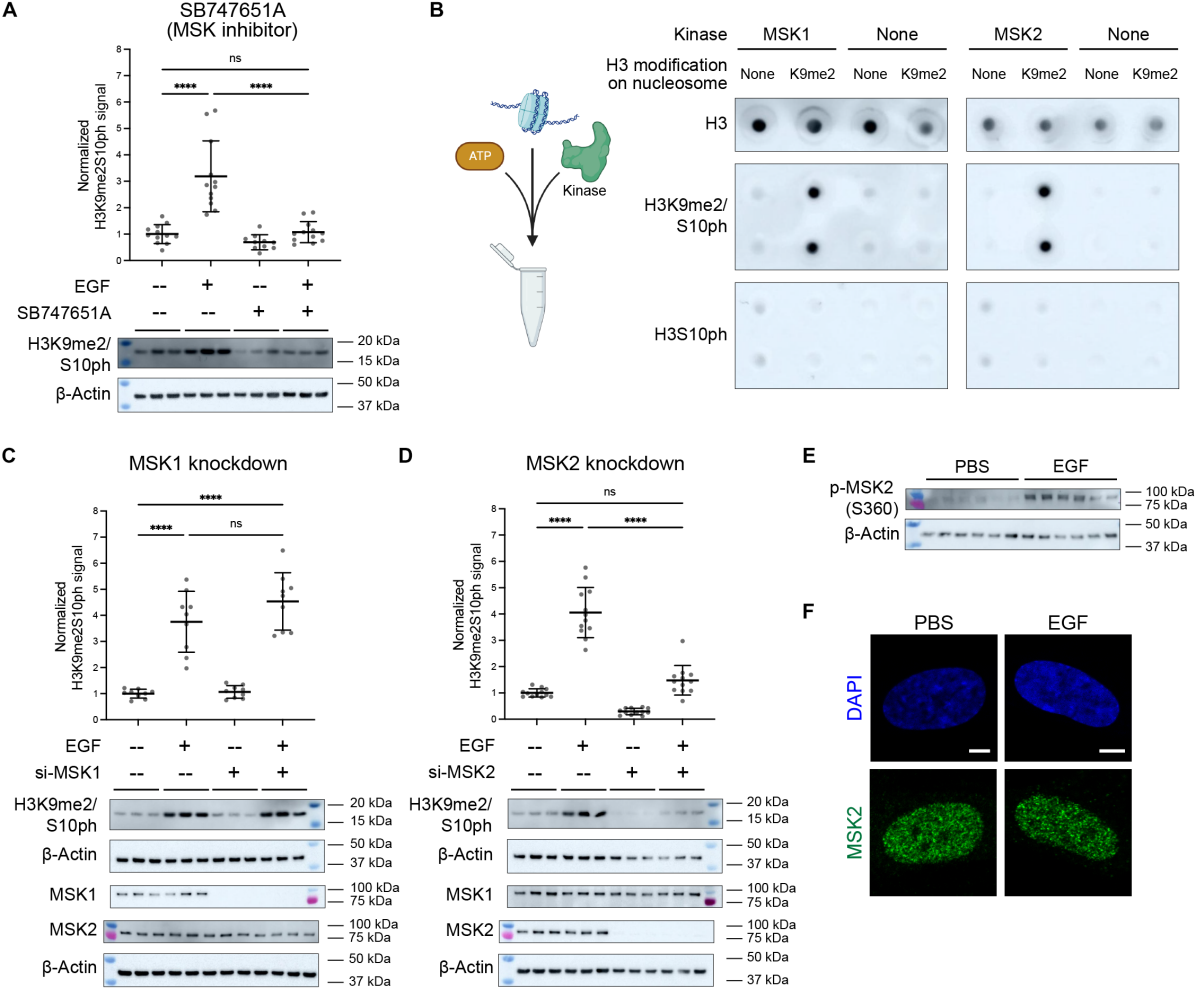
EGF induction of H3K9me2S10ph is MSK2 dependent. (**A**) Dot plot shows levels of H3K9me2S10ph measured by Western blots of serum-starved IMR90 cells treated with EGF or SB747651A compound (MSK inhibitor) as indicated. Measurements were normalized to loading control (β-actin) and the mean of the PBS sample. Lines indicate mean ± SD. *n* = 12 biological replicates per condition. Representative Western blots are shown below. (**B**) In vitro kinase assay in which active MSK1 or MSK2 was combined with ATP and recombinant nucleosomes with unmodified H3 tails (“None”) or dimethylated at Lys^9^ (“K9me2”). Immunoblots of each reaction were probed using antibodies to each histone modification as indicated. Schematic (left) created with BioRender. (**C**) Dot plot shows levels of H3K9me2S10ph measured by Western blots of serum-starved IMR90 cells treated with EGF and MSK1-targeting siRNA, as indicated. Measurements were normalized to loading control (β-actin) and the mean of the PBS sample. Lines indicate mean ± SD. *n* = 12 biological replicates per condition. Representative Western blots are shown below. (**D**) Dot plot shows levels of H3K9me2S10ph measured by Western blot from serum-starved IMR90 cells treated with EGF and MSK2-targeting siRNA, as indicated. Measurements were normalized to loading control (β-actin) and the mean of the PBS sample. Lines indicate mean ± SD. *n* = 9 biological replicates per condition. Representative Western blots are shown below. (**E**) Western blot for phospho-MSK2 (Ser^360^) and loading control (β-actin) from serum-starved IMR90 cells untreated (PBS) or treated with EGF for 30 min. (**F**) Representative confocal images of serum-starved IMR90 cells, untreated (PBS) or treated with EGF for 30 min, immunostained for MSK2 (green), and counterstained for DAPI (blue). Scale bars, 25 μm. Statistical analyses performed using two-way ANOVA. ****P < 0.0001.

EGF stimulation leads to a well-described signaling cascade that modulates numerous cellular responses by activating downstream kinases to phosphorylate transcription factors, histones, and other chromatin-interacting proteins (*29*–*31*). To identify a kinase responsible for EGF-dependent H3K9me2S10 phosphorylation, we tested the effect of different kinase inhibitors on H3K9me2S10ph levels in IMR90 cells (fig. S2A). Many of the inhibitors that reduced H3K9me2S10ph levels targeted kinases within the protein kinase A, G, and C (AGC) group, which includes the RSK/MSK kinase family (*32*). Kinases in this family have previously been shown to phosphorylate H3S10 (*33*–*35*). Given these results, we treated cells with SB747651A, a more specific inhibitor of the RSK/MSK family. Treatment of IMR90 cells with 5 μM SB747651A for 2 hours blocked the EGF-induced increase in H3K9me2S10ph (Fig. 2A).

It has been demonstrated that MSK1 phosphorylates H3S10 (*35*, *36*), but it has not been shown whether MSK1 or MSK2 can phosphorylate H3S10 when the adjacent residue is dimethylated (H3K9me2S10). We therefore tested the sufficiency of specific kinases to phosphorylate H3S10 in the context of nucleosomes with different modifications in an in vitro assay. Using nucleosome preparations in which the histone H3 tails were either unmodified (“None”) or dimethylated at the lysine-9 residue (“K9me2”), we mixed activated forms of MSK1 or MSK2 with adenosine triphosphate (ATP) and nucleosomes and assessed the resulting histone modifications by immunoblot. We found that both MSK1 and MSK2 were able to phosphorylate H3S10 when lysine-9 is dimethylated (Fig. 2B). Both kinases also showed a low but detectable ability to phosphorylate H3S10 when lysine-9 is unmodified. We cannot draw any conclusions regarding the relative activity of the kinases toward either substrate because we used different antibodies to detect H3S10ph or H3K9me2S10ph, but our data indicate that both MSK1 and MSK2 are able to phosphorylate H3K9me2S10.

Having demonstrated that both MSK1 and MSK2 are sufficient to phosphorylate H3K9me2S10 in vitro, we next asked whether either was necessary for H3K9me2S10 phosphorylation in response to EGF. We tested the impact of small interfering RNA (siRNA)–mediated knockdown of each kinase on the EGF-induced H3K9me2S10ph levels. While knockdown of MSK1 had no detectable effect on H3K9me2S10ph levels (Fig. 2C), MSK2 knockdown in IMR90 cells prevented the EGF-induced H3K9me2S10 phosphorylation (Fig. 2D). Neither MSK2 knockdown alone nor EGF stimulation in MSK2 knockdown cells changed the cell cycle profile of the cells (fig. S2B). Knockdown of MSK2 also prevented both the EGF- and PDGF-induced increase of H3K9me2S10ph in RPE1 cells (fig. S2, C and D).

To confirm that MSK2 can be activated by EGF stimulation, we treated cells with EGF for 30 min and performed Western blot for phospho-MSK2 (Ser^360^). The serine-360 residue of MSK2 is an autophosphorylation target required for activation of the N-terminal kinase domain, which phosphorylates downstream substrates of MSK2 (*37*, *38*). We observed an increase in phospho-MSK2 (Ser^360^) upon EGF stimulation, indicating that EGF can activate MSK2 in our system (Fig. 2E). Together, results of the MSK2 knockdown, in vitro kinase experiments, and assessment of EGF-induced activation led us to conclude that MSK2 is required for the cellular increase in H3K9me2S10ph following EGF stimulation in interphase.

To examine the cellular localization of MSK2, we performed IF and observed MSK2 primarily within the nucleus of both untreated and EGF-treated cells (Fig. 2F and fig. S2E). Its nuclear localization suggests that MSK2 acts downstream of canonical extracellular signal–regulated kinase (ERK) signaling in response to EGF, and consistent with this, the knockdown of MSK2 did not affect the activation of canonical EGF-responsive kinase cascade members such as ERK1/2, p90RSK, c-Raf, or MEK1/2 [mitogen-activated protein kinase (MAPK) kinase 1/2] (fig. S2F). Thus, although both MSK1 and MSK2 are capable of phosphorylating H3K9me2 on serine-10, MSK2 appears to be responsible for EGF-dependent stimulation of H3K9me2S10 in interphase IMR90 and RPE1 cells.

### A subset of EGF-induced transcriptional activation is MSK2 dependent

**Fig. 3.**
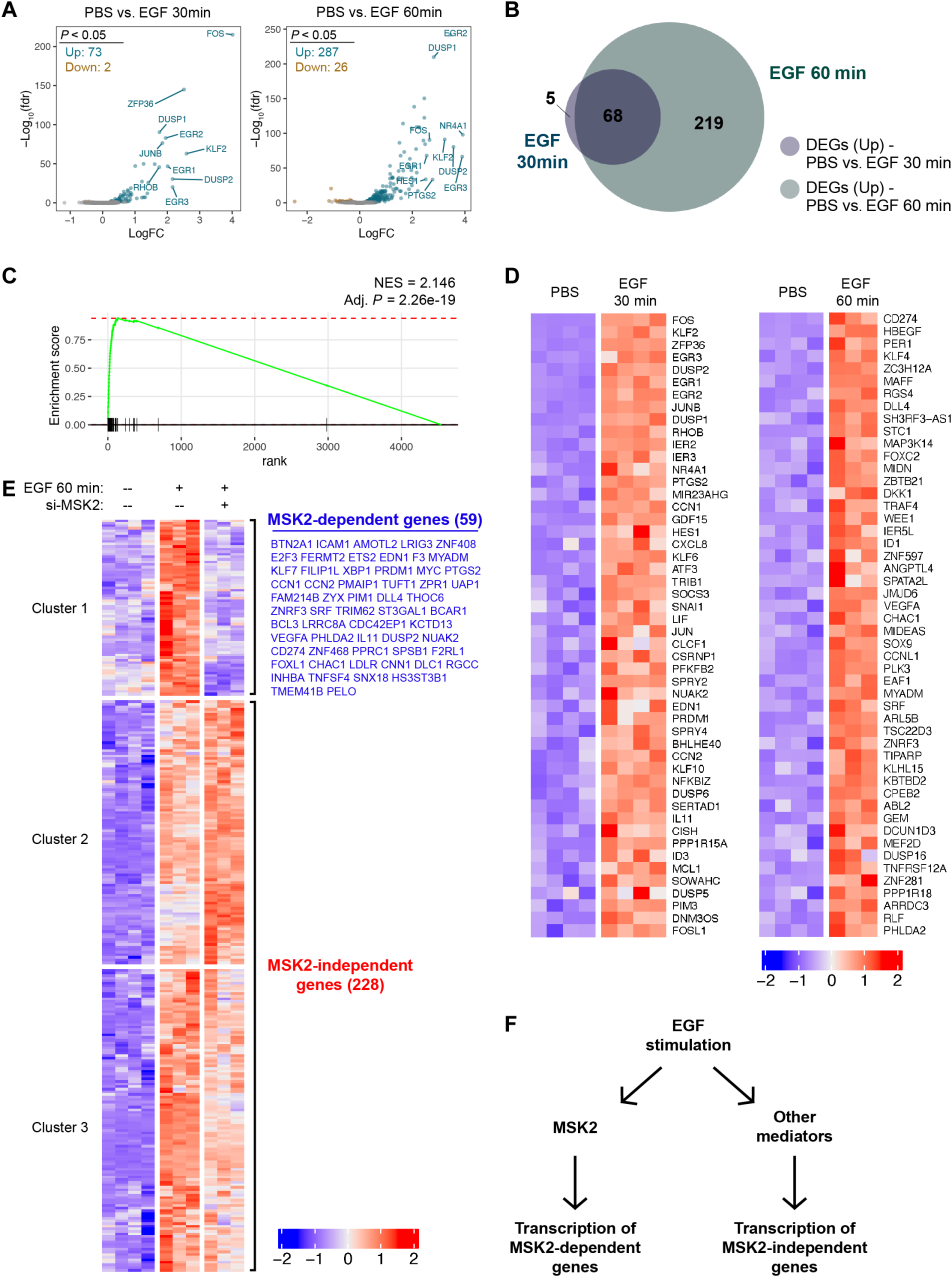
A subset of EGF-induced transcriptional activation is MSK2 dependent. (**A**) Volcano plots showing the differential gene expression patterns of serum-starved IMR90 cells treated with EGF for 30 or 60 min. Up-regulated genes (log fold change > 0.5; adjusted *P* < 0.05; green), down-regulated genes (log fold change < 0; adjusted *P* < 0.05; brown). (**B**) Venn diagram depicting the overlap of genes that are significantly up-regulated as measured after EGF treatment of 30 or 60 min. (**C**) GSEA enrichment plot on genes up-regulated after 60 min of EGF treatment compared to PBS in serum-starved IMR90 cells. Normalized enrichment score (NES) and adjusted *P* value are shown for the NAGASHIMA_EGF_SIGNALING_UP gene set in the C2 category of Molecular Signatures Database (MSigDB). (**D**) Heatmaps display normalized expression for differentially expressed genes (DEGs; adjusted *P* < 0.05 and ranked by descending log fold change). Left panel shows the top 50 DEGs after 30 min of EGF treatment. Right panel shows the top 50 DEGs unique to 60 min of EGF treatment. Colors are scaled by row in each heatmap. (**E**) Heatmap of all significant DEGs (adjusted *P* < 0.05) between PBS control, EGF 60-min treatment, and EGF 60-min treatment in cells with MSK2 knockdown. DEGs were grouped into clusters via hierarchical clustering based on gene expression across the three different conditions. The top cluster of genes was additionally tested for significant differential expression (adjusted *P* < 0.05) after 60 min of EGF treatment with or without MSK2 knockdown. These MSK2-dependent genes (*n* = 59) are labeled in blue next to the top cluster. Colors are scaled by row in each heatmap. (**F**) Schematic showing that a subset of EGF-stimulated transcription is activated through MSK2.

We next asked whether any EGF-induced gene expression changes were dependent on MSK2. To do this, we first determined which genes were altered in expression level in serum-starved IMR90 cells when treated with EGF. We isolated and sequenced RNA from untreated (PBS) cells and from cells following 30 or 60 min of EGF stimulation. Analysis of relative transcript abundance among these populations revealed significant changes in 75 genes after 30 min of EGF stimulation, 73 of which increased in expression. Many of these genes were canonical immediate-early response genes such as *FOS*, *JUNB*, and *EGR2* (Fig. 3, A and D, and table S1). After 60 min of EGF stimulation, 68 of these 73 genes were still significantly up-regulated, and an additional 219 genes were significantly up-regulated compared to the untreated samples (Fig. 3, B and D, and table S1). We performed gene set enrichment analysis (GSEA) and observed that the highest enrichment score corresponded with a dataset of EGF-stimulated gene expression (*39*) (normalized enrichment score = 2.146, adjusted *P* = 2.26 × 10^−19^; Fig. 3C), consistent with the differential gene expression signature that we expected.

We next interrogated the requirement of MSK2 for transcriptional changes of EGF-responsive genes by performing RNA sequencing on EGF-stimulated cells that were transfected with MSK2 siRNA. For the 287 genes that were significantly up-regulated after 60 min of EGF stimulation, we compared the transcript levels among the three conditions (untreated, EGF treated for 60 min, and EGF treated for 60 min with MSK2 knockdown). Hierarchical clustering of the datasets revealed that one of the clusters contained genes that were activated by EGF but were not similarly activated in the setting of MSK2 knockdown. Within that cluster, we confirmed that the EGF-induced expression of 59 genes was significantly prevented by MSK2 knockdown, and we termed these MSK2-dependent genes (Fig. 3, E and F, and table S2). The remaining 228 EGF-induced genes were designated MSK2-independent genes (Fig. 3, E and F, and table S2).

### EGF stimulation induces locus-specific increases of H3K9me2S10ph

**Fig. 4.**
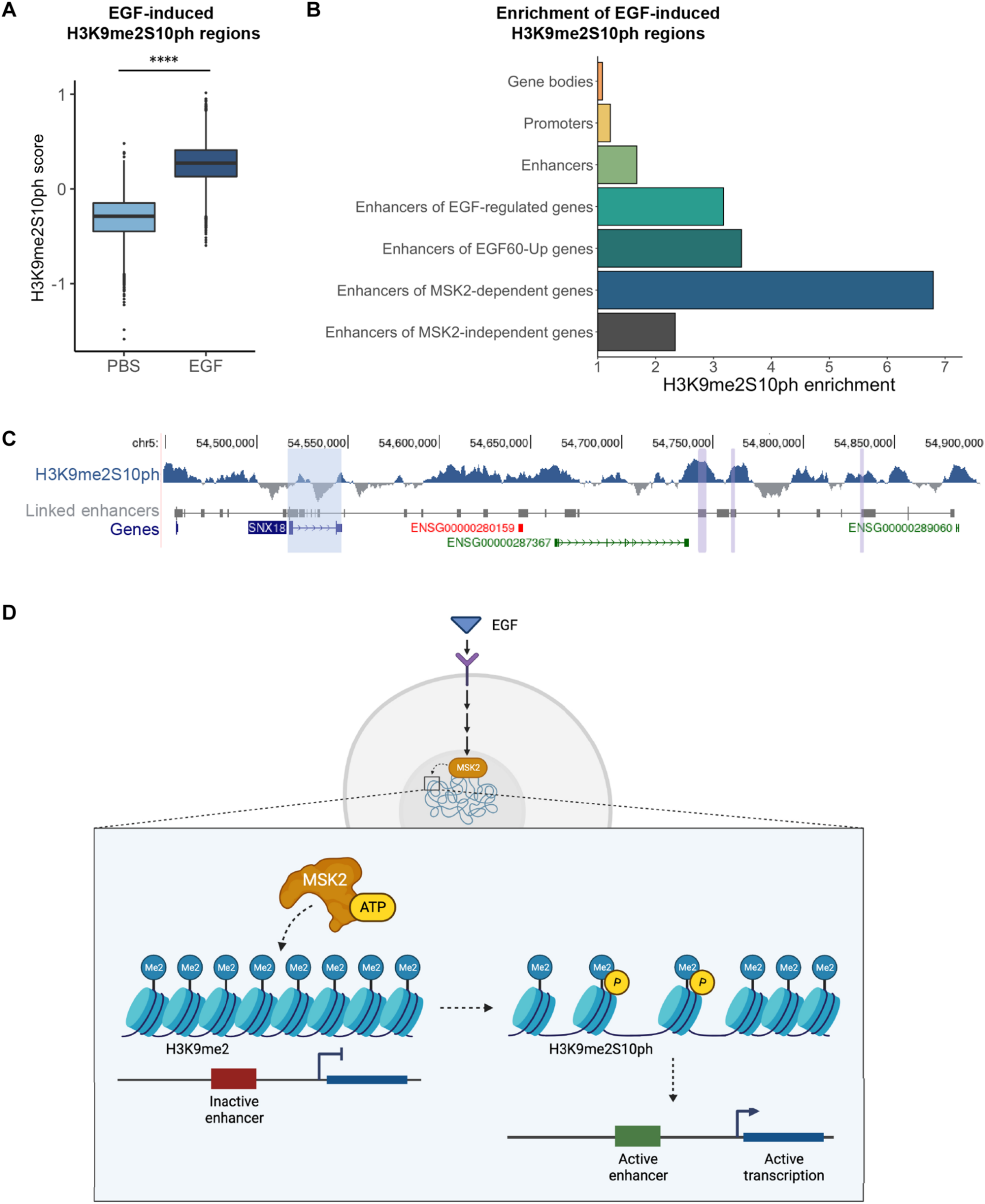
EGF stimulation induces locus-specific increases of H3K9me2S10ph. (**A**) Distributions of average H3K9me2S10ph *z* scores in chromatin regions that were significantly enriched (adjusted *P* < 0.005) for H3K9me2S10ph in a comparison between IMR90 cells that were untreated (PBS) or treated with EGF for 30 min. Boxplots show median, 25% and 75% quartiles. (**B**) Enrichment (fold change of observed over expected overlap for randomly permuted genomic regions of equal sizes) of EGF-dependent H3K9me2S10ph regions for each annotation category. Categories as defined in Fig. 3: “Enhancers of EGF-regulated genes” refers to the complete set of 320 genes that were significantly differentially expressed after either 30 or 60 min of EGF stimulation; “Enhancers of EGF60-Up genes” refers to the 219 genes uniquely up-regulated after 60 min of EGF stimulation; “Enhancers of MSK2-dependent genes” refers to the 59 genes defined as MSK2 dependent; “Enhancers of MSK2-independent genes” refers to the 228 genes defined as MSK2 independent. All enrichment analyses were subjected to 1000 simulations; all *P* values < 0.001. (**C**) UCSC Genome Browser view (hg38) showing EGF-dependent H3K9me2S10ph in a region around the EGF-regulated and MSK2-dependent gene SNX18 (blue highlight). Linked Enhancers track shows GeneHancer annotations of SNX18-associated enhancers (gray boxes). Purple highlights indicate significant EGF-dependent H3K9me2S10ph regions [as in (A) and (B)] that overlap annotated enhancers. (**D**) Schematic of proposed model: EGF stimulation leads to the activation of the nuclear kinase MSK2, which mediates H3K9me2S10 phosphorylation at enhancers of EGF-responsive genes, leading to transcriptional activation. Schematic created with BioRender.

The observation that growth factor stimulation increases the overall abundance of H3K9me2S10ph led us to ask which regions of the genome were specifically marked with this histone modification after EGF stimulation. Therefore, we assayed genome-wide distributions of H3K9me2S10ph in untreated IMR90 cells and in cells that were treated with EGF for 30 min [using cleavage under target and release using nuclease (CUT&RUN), see Materials and Methods]. We found 9206 genomic regions in which the H3K9me2S10ph signal was significantly higher (adjusted *P* < 0.005) in IMR90 cells following EGF stimulation compared to the untreated samples (Fig. 4A). These regions were an average of 1.5 kb and cover, in total, approximately 0.03% of the mappable human genome.

To determine whether these H3K9me2S10ph-modified regions were biologically meaningful, we asked if they were randomly distributed in the genome or nonrandomly distributed and overlapped annotated genome features more often than expected by chance. For each comparison, we performed 1000 random permutations to estimate the probability of a given observation happening by chance, given the sizes of the regions and the genome (see Materials and Methods). When we compared the EGF-induced H3K9me2S10ph-modified regions with the locations of known genome elements, we found that H3K9me2S10ph-modified sites were enriched at all annotated distal transcriptional enhancers (*40*) by 1.68-fold observed over expected (Fig. 4B). Further, at the subset of distal transcriptional enhancers associated with the EGF-regulated genes defined by our RNA-sequencing analysis (see Fig. 3), H3K9me2S10ph-modified regions were over 3-fold (3.17-fold) more likely than expected by chance to overlap those enhancers (Fig. 4B). The enrichment was even greater, 3.48-fold, at enhancers of genes that increased in transcription after 60 min of EGF stimulation (“Enhancers of EGF60-Up genes”; Fig. 4B). In contrast, regions with increased H3K9me2S10ph after EGF stimulation did not overlap gene bodies more frequently than expected by chance (1.08-fold observed over expected), and only overlapped promoters slightly more frequently than expected (1.21-fold). The most striking enrichment of H3K9me2S10ph-modified regions was seen for enhancers of the MSK2-dependent genes (see Fig. 3E), which were 6.8-fold more likely than expected (Fig. 4, B and C). Enhancers of MSK2-independent genes had notably lower enrichment (2.34-fold) of H3K9me2S10ph-modified regions (Fig. 4B). The distribution of H3K9me2S10ph-modified regions across the genome suggests that this histone modification is associated with the activity state of some transcriptional enhancers.

## DISCUSSION

Classic studies of growth factor signaling pathways have largely focused on activation of transcription factors and changes in gene expression. Here, we show that growth factor stimulation of interphase cells leads to an increase in H3K9me2S10 phosphorylation levels and that, in EGF-stimulated IMR90 cells, locus-specific changes in H3K9me2S10ph frequently occur at the enhancers of EGF-responsive genes. We identify a nuclear kinase, MSK2, as an EGF signal effector. MSK2 phosphorylates H3K9me2-modified nucleosomes at the adjacent serine-10 residue in vitro and is required for a subset of EGF-induced transcriptional changes. Our results indicate that downstream consequences of EGF stimulation include MSK2-mediated phosphorylation of H3K9me2S10 at the enhancers of EGF-responsive genes, concordant with these genes becoming permissive for transcriptional activation (Fig. 4D).

The observation that EGF stimulation results in an increase in the cellular abundance of H3K9me2S10ph combined with the ability of MSK2 to phosphorylate H3K9me2 in vitro led us to propose a model in which increased H3K9me2S10ph arises through phosphorylation of H3K9me2 chromatin at S10 (rather than dimethylation of preexisting H3S10ph). EGF-induced H3K9me2S10ph occurs at the enhancers of EGF-induced genes, so these enhancers were likely inactive before stimulation and may have been located within H3K9me2-marked chromatin. H3K9me2 is one of the most widespread chromatin modifications in eukaryotic cells and is associated with the organization of heterochromatin at the nuclear periphery (*7*, *24*). While most H3K9me2-modified domains overlap with lamin-associated domains (LADs) (*7*), a smaller subset of H3K9me2-marked chromatin that does not associate with the nuclear lamina (known as KODs) is enriched for inactive transcriptional enhancers (*26*). The increase in H3K9me2S10ph was assessed at 30 min after EGF stimulation, while many of the corresponding transcriptional changes were first detected 60 min after EGF stimulation, raising the possibility that the presence of H3K9me2S10ph at enhancers may precede transcriptional activation. Given the relatively low abundance of H3S10ph in interphase cells, as assessed by both Western blot and mass spectrometry (Fig. 1, E, G, and H), it is unlikely that H3K9me2S10ph arises through H3S10ph-modified chromatin subsequently being dimethylated on lysine-9, although we cannot formally exclude this possibility. Therefore, we propose that the EGF-stimulated increase in H3K9me2S10ph is most likely a result of phosphorylation of H3K9me2-marked chromatin at serine-10.

Previous work has demonstrated that activation of genes within H3K9me2-modifed domains corresponds with loss of H3K9me2 and movement of relevant genomic loci from the nuclear periphery to the nuclear interior (*7*, *25*), but the molecular mechanism of H3K9me2-marked chromatin detachment from the nuclear periphery is unknown. While this study did not examine the positions of EGF-responsive loci relative to the nuclear lamina, one hypothesis is that the phosphorylation of H3S10 adjacent to H3K9me2 acts as a phospho-methyl switch to facilitate detachment of H3K9me2-marked heterochromatin from the nuclear periphery to enable transcriptional activation. This is based on observations in mitotic cells where histone phospho-methyl switches have been proposed to displace or prevent the association of chromatin-binding proteins to specific histone modifications, or to detach and repel chromatin from the nuclear lamina at the onset of mitosis (*14*–*17*, *24*). Since we observe a peak in the abundance of H3K9me2S10ph at 30 min after EGF, H3K9me2S10ph might represent a transient state that facilitates the detachment of H3K9me2 chromatin from the nuclear periphery during interphase as a result of growth factor–triggered kinase activity. Future studies will aim to test this hypothesis and to determine if H3K9me2S10 phosphorylation might render targeted enhancers accessible for a limited period of time after exposure to an extracellular signal, allowing for subsequent gene activation.

While we have shown that some EGF-induced genes require MSK2 for induction of transcription, we did not observe H3K9me2S10ph increases at the enhancers of all EGF-responsive genes, nor were all EGF-responsive genes transcriptionally affected by the knockdown of MSK2. This indicates that MSK2 is only responsible for a subset of EGF-induced transcriptional activation and that other modes of EGF responses, such as mechanisms of classical signaling through transcription factor activity, also occur (*41*, *42*). It is possible that the enhancers regulating MSK2-dependent genes are found within H3K9me2-marked chromatin before EGF stimulation, and the MSK2-mediated phosphorylation of H3K9me2S10 contributes to these regions becoming accessible for transcriptional activation, while EGF-responsive genes found in non–H3K9me2-marked regions undergo a different mode of activation. It is also unclear whether MSK2 acts independently or in a complex with other adapter and effector proteins, the various combinations of which may influence their genomic targets. Important areas for future study include examining changes in H3K9me2 patterns, as well as identifying other possible differences between MSK2-dependent and MSK2-independent enhancers such as their nuclear positioning before EGF stimulation. The results of such experiments will help to clarify the chromatin-related mechanisms of growth factor signal–mediated gene activation.

H3S10ph and H3K9me2 have been proposed to be mutually exclusive histone modifications, and the presence of each mark has been shown to prevent the “spreading” of the other into adjacent genomic regions (*43*, *44*). However, few studies in mammalian cells have explored possible distinctions between H3S10ph and H3K9me2S10ph. This may also be due to the fact that many commercial antibodies that recognize H3S10ph lose that recognition when adjacent residues, including Lys^9^, are also modified (fig. S1D). Early studies treating serum-starved mouse cells with various mitogens and growth factors demonstrated histone serine phosphorylation within a few minutes of stimulation (*1*), but lacked the resolution to identify additional modifications. Another study confirmed that H3S10 was phosphorylated in response to mitogen stimuli and EGF pathway activation in fibroblasts, and further demonstrated that MSK2 was the major kinase for this event (*35*). H3S10ph was observed by IF in heterochromatin regions in serum-starved cells, which raises the possibility that these MSK2-dependent H3 phosphorylation events were, in fact, H3K9me2S10ph. A number of recent reports also describe growth factor–induced H3S10 phosphorylation at promoters due to MSK1 or PIM1 (*45*–*47*). This is in contrast to our observation of H3K9me2S10ph being much more significantly enriched at enhancers of EGF-responsive genes due to MSK2. These combined results could highlight one distinction between H3K9me2S10ph and H3S10ph, as well as distinguishing functions between MSK1 and MSK2. Additionally, both H3S10ph and H3K9me2S10ph are present in low abundance in interphase cells. By mass spectrometry in G_1_-G_0_ serum-starved cells, H3K9me2 is ~280 times more abundant than H3K9me2S10ph and ~850 times more abundant than H3S10ph (Fig. 1H). While we detected an increase in cellular H3K9me2S10ph but not H3S10ph following EGF stimulation of IMR90 cells, it is possible that the locus-specific increases in H3S10ph reported in previous studies (*45*–*47*) occur in small enough amounts that they were not detectable in our population-based assays.

While numerous studies have focused on kinases that target H3S10, H3K9me2S10 has not been examined as a kinase substrate to the same extent. The closest *Drosophila* ortholog to MSK2, JIL-1, has been implicated as an H3K9me2S10 kinase. JIL-1 was shown to phosphorylate both H3S10 and H3K9me2S10 in interphase cells to facilitate gene expression (*48*–*50*). JIL-1 is predominantly localized to euchromatin, and its depletion was shown to cause genome-wide H3K9me2 spreading into previously euchromatic regions, suggesting that JIL-1 acts to maintain open chromatin regions that facilitate gene expression (*44*, *51*). Although the effects of MSK2 seem to be more localized, it may fulfill a similar role as JIL-1 to promote accessible chromatin for transcription.

It is currently unclear how histone-modifying kinases, including MSK2, are directed to phosphorylate specific locations in the genome. Recent work has demonstrated that JIL-1 is targeted to chromatin through its association with an adapter protein JASPer, which contains a PWWP domain with an aromatic pocket that binds methyl-lysines (*52*, *53*). While an adapter protein for MSK2 has yet to be identified, JASPer shares homology with the human protein LEDGF/p75 (*52*), which could be examined in the future for MSK2 adapter functions. While LEDGF/p75, or another adapter, might help to direct MSK2 to H3K9me2 regions, it remains to be determined what mechanisms provide locus specificity for MSK2 targeting to growth factor–regulated enhancers.

## MATERIALS AND METHODS

### Cell culture

Human fetal lung fibroblast cell line IMR90 was obtained from the American Type Culture Collection (ATCC) (#CCL-186) and maintained at 37°C in DMEM (#10-013-CV, Corning) supplemented with 10% fetal bovine serum (FBS) (#S11150, Atlanta Biologicals). hTERT-immortalized human retina pigmented epithelial cell line RPE1 was originally obtained from ATCC (#CRL-4000) and maintained at 37°C in Dulbecco’s modified Eagle’s medium (DMEM)/F12 with GlutaMAX (#10565018, Thermo Fisher Scientific) supplemented with 10% FBS and hygromycin B (0.01 mg/ml). Cells were serum-starved by removing medium, rinsing cells with base medium (no FBS or hygromycin B), and incubating at 37°C in base medium for 22 to 26 hours. All cell lines were routinely tested for mycoplasma contamination.

For EGF stimulation experiments, recombinant human EGF (#PRD236-50, R&D Systems) was reconstituted in 1× PBS and added to cell culture medium to a final concentration of 750 pM for 30 min unless otherwise stated. For PDGF stimulation experiments, recombinant human PDGF (#1159-SB-025, R&D Systems) was reconstituted in HCl and added to cell culture medium to a final concentration of 7.143 μM PDGF and 4 μM HCl for 30 min.

### Cell cycle characterization

Cell cycle was assessed using flow cytometry for propidium iodide (Cell Cycle Kit; #C03551, Beckman Coulter). Between 500,000 and 1 million cells were washed twice in 1× Dulbecco’s PBS (DPBS), fixed in 70% ethanol at −20°C for 1 hour, and then incubated in 500 μl of propidium iodide at room temperature in the dark for 15 min. Flow cytometry analysis was performed on a LSR II (BD Biosciences).

### Immunofluorescence

IF assays were performed as previously described (*24*, *54*). IMR90 and RPE1 cells used for imaging experiments were grown on glass coverslips and fixed with 4% paraformaldehyde (#15710, Electron Microscopy Sciences) for 10 min at room temperature, washed three times with DPBS (#21-031-CV, Corning), and permeabilized with 0.25% Triton X-100 (#28314, Thermo Fisher Scientific) for 10 min. Cells were then blocked in 1% bovine serum albumin (BSA) (#A4503, Sigma-Aldrich) in PBS with 0.05% Tween 20, pH 7.4 (#28320, Thermo Fisher Scientific) for 60 min. Primary antibodies diluted in 1% BSA/PBS–Tween 20 (PBS-T) were added for 60 min and then washed three times in PBS-T for 5 min. Secondary antibodies diluted in 1% BSA/PBS-T were added for 60 min and then washed twice with PBS-T and once with PBS for 5 min. Samples were mounted using Duolink In Situ Mounting Medium with 4′,6-diamidino-2-phenylindole (DAPI) (#DUO82040-5ML, Sigma-Aldrich).

### Image acquisition and analysis

Confocal immunofluorescent images were taken using a Leica SP8 laser scanning confocal system using 63×/1.40 HC PL APO CS2 objective and HyD detectors in the standard mode with 100% gain. Images for quantitative analysis were taken with similar parameters. Image analysis was performed using ImageJ software (National Institutes of Health, MD). Analysis of H3K9me2S10ph and H3S10ph levels in cells treated with EGF, PDGF, or control cells was performed by measuring the total fluorescence intensities of H3K9me2S10ph and H3S10ph in individual nuclei using a mask created based on nuclear lamina staining (lamin A/C or lamin B1). Analysis of MSK2 distribution in cells treated with EGF and control cells was performed by measuring the total fluorescence intensities of MSK2 in the nucleus and in the whole cell using DAPI signal and total MSK2 signal to create masks for the nucleus and the whole cells, respectively. The fraction of MSK in the nucleus was calculated as a ratio of MSK2 signal in the nucleus to total MSK2 signal in the cell. Analysis of H3K9me2S10ph in interphase cells treated with EGF and control cells was performed by measuring the total fluorescence intensities of H3K9me2S10ph and Aurora B in individual nuclei using a mask created based on DAPI signal. Cells with low Aurora B signal were treated as interphase cell, while cells with high Aurora B signal (a marker of mitotic entrance) were marked as “mitotic” and excluded from the analysis.

### Western blot analysis

Whole-cell protein lysates were collected in 1× radioimmunoprecipitation assay (RIPA) buffer, mixed with sample buffer and reducing agent, heated at 95°C for 5 min, and then run on a 4 to 12% bis-tris protein gel. See Table 1 for a list of reagents used. Western blot signal was quantified in ImageJ by measuring integrated density over equivalent areas per sample. Measurements were normalized by dividing over the average of the vehicle control condition.

**Table 1.** List of reagents used for Western blot experiments.

Reagent	Vendor	Catalog number	Application
10× RIPA buffer	Cell Signaling Technology	#9860S	RIPA buffer component
10% SDS	Thermo Fisher Scientific	#15553-035	RIPA buffer component
Sodium butyrate	MilliporeSigma	#B5887	RIPA buffer component
Benzonase nuclease	MilliporeSigma	#E1014	RIPA buffer component
Halt 100× protease and phosphatase inhibitor	Thermo Fisher Scientific	#78442	RIPA buffer component
NuPAGE MES SDS running buffer (20×)	Invitrogen	#NP0002	Immunoblot protein gel
NuPAGE 4–12% bis-tris gel, 1.5 mm × 15 well	Invitrogen	#NP0323	Immunoblot protein gel
4× Laemmli sample buffer	Bio-Rad	#161-0747	Immunoblot protein gel
10× Reducing agent	Invitrogen	#NP0009	Immunoblot protein gel
Precision plus protein standards	Bio-Rad	#161-0374	Immunoblot protein gel
NuPAGE transfer buffer (20×)	Invitrogen	#NP0006	Immunoblot protein transfer
Nitrocellulose membrane filter paper sandwich	Invitrogen	#LC2000	Immunoblot protein transfer
Bovine serum albumin, low IgG powder	Gemini	#700-105P	Immunoblot blocking buffer
Amersham ECL prime detection reagent	Cytiva Life Sciences	#RPN2232	Immunoblot detection
Restore Western blot stripping buffer	Thermo Fisher Scientific	#21059	Immunoblot stripping

### Histone posttranslational modification analysis by quantitative mass spectrometry

Coomassie staining was performed to confirm the enrichment of histones. Purified histones starting with 5 to 20 μg of estimated protein were used for chemical derivatization and digestion as described previously (*55*). Unmodified lysines were derivatized twice with a 1:3 ratio of acetonitrile to proprionic anhydride. Histones were then digested with trypsin in a 1:20 enzyme to protein ratio at 37°C overnight. Digested histones with newly formed N termini were derivatized twice as performed before. Finally, histones were dried with a vacuum concentrator. The dried samples were reconstituted in 0.1% trifluoroacetic acid (TFA) and desalted with the C18 micro spin column (Harvard Apparatus). The column was prepared with 200 μl of 100% acetonitrile and equilibrated with 200 μl of loading buffer with 0.1% TFA. Peptides were loaded onto the column, washed with loading buffer, and eluted with 200 μl of 70% acetonitrile in 0.1% formic acid. All steps for loading, washing, and elution were carried out with benchtop centrifugation (300*g* for 2 min). The eluted peptides were then dried with a vacuum concentrator.

Dried histone peptides were reconstituted in 0.1% formic acid. A synthetic library of 93 heavy labeled and derivatized peptides containing commonly measured histone posttranslational modifications (*56*) was spiked into the endogenous samples to a final concentration of approximately 100 ng μl^−1^ for endogenous peptides and 100 fmol μl^−1^ for each heavy labeled synthetic analyte. For each analysis, 1 μl of sample was injected onto the column for data-independent analysis on a Q-Exactive HF instrument (Thermo Fisher Scientific) attached to an Ultimate 3000 nano-UPLC system and Nanospray Flex ion source (Thermo Fisher Scientific). Using aqueous solution of 0.1% formic acid as buffer A and organic solution of 80% acetonitrile and 0.1% formic acid as buffer B, peptides were separated on a 63-min gradient at 400 nl min^−1^ starting at 4% buffer B and increasing to 32% buffer B over 58 min and then increasing to 98% buffer B over 5 min. The column was then washed at 98% buffer B over 5 min and equilibrated to 3% buffer B. Data-independent acquisition was performed with the following settings. A full MS^1^ scan from 300 to 950 mass/charge ratio (*m*/*z*) was acquired with a resolution of 60,000, an automatic gain control (AGC) target of 3 × 10^6^, and a maximum injection time of 55 ms. Then, a series of 25 MS^2^ scans was acquired across the same mass range with sequential isolation windows of 24 *m*/*z* with a collision energy of 28, a resolution of 30,000, an AGC target of 1 × 10^6^, and a maximum injection time of 55 ms. Data analysis and manual inspection using the synthetic library as a reference were performed with Skyline (MacCoss Lab). Ratios were generated using R Studio.

### Antibody specificity dot blots

Histone H3 tail peptides (amino acids 1 to 20, synthesized by G. Burslem at the University of Pennsylvania) with indicated modifications were spotted (1 and 0.5 μg) on nitrocellulose and allowed to dry. Membranes were then probed with antibodies as for standard Western blots.

### In vitro kinase assay

For each reaction, histone kinase assays were performed using 1 μg of recombinant human biotinylated nucleosomes (unmodified: #116-0006, H3K9me2: #16-0324-20, Epicypher) in 30 μl of kinase buffer [8 mM Mops/NaOH (pH 7.0), 0.2 mM EDTA, 10 mM MgAc, 0.1 mM ATP]. Enzyme amounts used per kinase reaction were 30 ng (0.016 U) of MSK1 (#14-548, Millipore) and 95 ng (0.015 U) of MSK2 (#14-616, Millipore), based on the manufacturer’s determined specific activity of each enzyme lot. Reactions were stopped after 10 min at 30°C by adding Laemmli sample buffer (Bio-Rad). Reactions were spotted on nitrocellulose (Bio-Rad) and processed for immunoblotting with antibodies indicated.

### Kinase chemical inhibition and siRNA knockdown

Inhibitor experiments were performed by treating cells at 37°C for 2 hours with the inhibitors and doses specified in Table 2.

**Table 2.** List of chemical compounds used for kinase inhibition experiments.

Compound	Doses used	CAS number	Vendor
H-89	0, 500 nM, 1 μM, 2 μM, 20 μM, 30 μM, 50 μM, 70 μM	127243-85-0	MilliporeSigma
ML-7	0, 500 nM, 1 μM, 2 μM, 20 μM, 30 μM, 50 μM, 70 μM	110448-33-4	MilliporeSigma
W-7	0, 1 μM, 2 μM, 20 μM, 30 μM, 50 μM, 70 μM	61714-27-0	MilliporeSigma
Staurosporine	0, 10 nM, 20 nM, 50 nM	62996-74-1	MilliporeSigma
KN-93	0, 2 μM, 20 μM, 50 μM, 100 μM, 500 μM	139298-40-1	MilliporeSigma
Bisindolylmaleimide I	0, 100 nM, 500 nM, 1 μM	176504-36-2	MilliporeSigma
PKG inhibitor	0, 50 μM, 100 μM	Peptide competitive inhibitor (sequence: RKRARKE)	MilliporeSigma #370654
IKK-16	0, 100 nM, 200 M, 500 nM, 1 μM, 10 μM, 20 μM	1186195-62-9	MilliporeSigma
SB747651A	5 μM	1781882-72-1	Tocris Bioscience

Knockdown experiments used ON-TARGETplus siRNA from Horizon Discovery (Cambridge, UK) against the targets in Table 3. siRNA transfection was performed using Lipofectamine RNAiMAX (#13778-075, Thermo Fisher Scientific) according to the manufacturer-provided protocol. A final concentration of 10 nM siRNA was added to cell culture medium every 24 hours for 72 total hours of incubation.

**Table 3.** List of siRNAs used for knockdown experiments.

Gene	Accessions targeted		Catalog number
Non-targeting control	n/a		#D-001810-10-20
RPS6KA4 (MSK2)	NM_001006944 NM_001300802 NM_001318361	NM_003942 XM_005274380 XM_017018527	#L-004664-00
RPS6KA5 (MSK1)	NM_001322227 NM_001322228 NM_001322229 NM_001322230 NM_001322231 NM_001322232 NM_001322233 NM_001322234	NM_001322235 NM_001322236 NM_001322237 NM_001322238 NM_004755 NM_182398 XM_017021786	#L-004665-00

### RNA isolation, library preparation, and sequencing analysis

Total RNA was collected using QIAGEN RNeasy kit. First-strand cDNA synthesis and amplification were performed using the SMART-Seq v4 Ultra Low Input RNA Kit (#634890, Takara Bio). Sequencing libraries were assembled using Nextera XT DNA Library Preparation Kit (#FC-131-1024, Illumina). Samples were sequenced on an Illumina NextSeq 550 platform with 2 × 35 base pairs and 75 total cycles (#20024906, Illumina).

Sequences were demultiplexed using bcl2fastq2 (v2.20.0.422) and aligned to hg38 reference genome using STAR (v2.7.9a). Aligned reads were then counted using HTseq (v0.13.5). Downstream analyses were conducted in R (v4.1.2) using edgeR (*57*–*59*) (v3.36.0). Genes were filtered to retain genes that had a counts per million value greater than 10 in at least two samples. Negative binomial generalized linear models for common, trended, and tagwise dispersion were fitted using default parameters. Exact tests [edgeR function exactTest()] with the auto-selected dispersions were performed between any given two groups followed by multiple test correction using the Bonferroni-Hochberg method. Heatmaps were made using ComplexHeatmap (v2.10.0) (*60*, *61*) with RPKM (reads per kilobase per million) normalized counts. GSEAs were performed using fgsea (v1.20.0) and msigdbr (v7.5.1) packages for the “C2” (Curated Gene Sets) category from the Molecular Signatures Database (*62*–*64*). Additional plots were made using ggplot2 and patchwork packages.

### Cleavage under target and release using nuclease

CUT&RUN was performed following the CUTANA CUT&RUN kit v3.5 protocol (#14-1048, Epicypher). Per reaction, between 250,000 and 500,000 cells and 1 μl of H3K9me2S10ph antibody (Millipore, #05-1354) or 0.5 μl of mouse immunoglobulin G (IgG) antibody (Cell Signaling, #5415) were used.

### Library preparation for CUT&RUN

Sequencing libraries were prepared for next-generation sequencing using the KAPA HyperPrep Kit (#KK8502 07962347001, Kapa Biosystems) and the NEBNext Multiplex Oligos for Illumina (#E6440S, New England Biolabs) with modifications as previously described (*23*). Briefly, the end-repair and A-tailing temperature was dropped from 65°C to 58°C, and the reaction time for this step was increased to 60 min. Adapter ligation was performed using the NEB adapter stock diluted to the desired concentration. Following adapter ligation, the manufacturer-recommended volume of NEB USER enzyme was added to the reaction and incubated at 37°C for 15 min. Cleanup after adapter ligation and hairpin cleavage was performed using 1.1× Agencourt AMPure Beads (#A63880, Beckman Coulter). For the amplification reaction, 10 μl of the NEBNext Multiplex Oligos was added to the samples, replacing the KAPA Library Amplification Primer Mix, and subjected to 13 to 14 cycles of amplification. Post-amplification cleanup was carried out using 1.1× Agencourt AMPure Beads. Libraries were quantified with Qubit 2.0 using the DNA High Sensitivity kit (#Q32854, Thermo Fisher Scientific), and library quality and average fragment size were determined with TapeStation using the High Sensitivity D5000 ScreenTape (#5067-5592, Agilent Technologies) and reagents (#5067-5593, Agilent Technologies). Libraries were pooled together in equimolar ratio and sequenced on an Illumina NextSeq 1000 platform with 2 × 42 base pairs and 100 total cycles (#20046811, Illumina).

### Sequencing analysis for CUT&RUN

Paired-end reads were demultiplexed using bcl2fastq (v2.20.0.422) and aligned to hg38 reference genome using Bowtie2 (v2.3.5.1) (*65*, *66*) with parameters “—very-sensitive-local –no-unal –no-mixed –no-discordant -I 10 -X 1000.” Duplicated reads were annotated using Picard MarkDuplicates, and ENCODE denylist (hg38, v2) (*67*) regions were removed using bedtools (v2.26.0) (*68*) function intersect. Visualization tracks for each library were generated using samtools (v1.17) to sort and index, and then deepTools (v3.5.1) function “bamCoverage” with counts per million normalization and parameters “—samFlagExclude 1548” to exclude unmapped, unpaired, duplicate, and failed QC reads. IgG normalization (CUT&RUN) was performed using deepTools function “bigwigCompare” with operation “subtract.”

To generate read coverage in fixed windows, the genome was binned into 1-kb bins, and the normalized read count was computed for each bin using deepTools function multiBigwigSummary. Samples of each condition were converted to *z* scores to account for small differences in dynamic range between experiments. *z* scores were then smoothed using the mean of rolling, centered windows of size 10 to produce final scores in 1-kb bins. Adjacent bins were merged using bedtools (v2.29.2) (*68*) function merge (maximum distance between features, 0) to assign individual domains for further analyses.

Statistical significance of differential H3K9me2S10ph enrichments was determined by performing a Student’s *t* test for two-way comparisons across replicates (PBS versus EGF). We corrected the *P* values of the *t* test using the Benjamini-Hochberg correction for multiple testing (*69*) and determined significant enrichments with an adjusted *P* value threshold of <0.005.

### Statistical test for enrichment

Statistical analyses for enrichment were performed using Genomic Association Tester (GAT) (*70*). The significance of overlap between sets of genomic intervals was calculated on the basis of simulation using a permutation-based approach and accounting for genome organization regions of low mappability. All enrichment analyses were subjected to 1000 simulations. The fold enrichment is expressed as the ratio of observed/expected. *P* values reflect an estimate of the probability to obtain an observed (or larger) overlap between two segment sets by chance.

### Antibodies

The antibodies listed in Table 4 were used.

**Table 4.** List of antibodies used in this study.

Antibody target	Vendor	Catalog number	Application
H3K9me2S10ph	Millipore Sigma	05-1354	Immunofluorescence, Western blot, antibody specificity dot blot, in vitro kinase assay, CUT&RUN
H3S10ph	Active Motif	39253	Immunofluorescence, Western blot, antibody specificity dot blot, in vitro kinase assay
H3K9me2	Active Motif	39239	Antibody specificity dot blot
H3K9me3	Abcam	ab8898	Antibody specificity dot blot
Histone H3	Abcam	ab1791	Western blot, antibody specificity dot blot, in vitro kinase assay
Aurora B kinase	Abcam	ab2254	Immunofluorescence
Lamin B1	Abcam	ab16048	Immunofluorescence
Donkey anti-mouse, Alexa Fluor 488	Thermo Fisher Scientific	A21202	Immunofluorescence
Donkey anti-mouse, Alexa Fluor 568	Thermo Fisher Scientific	A10037	Immunofluorescence
Donkey anti-rabbit, Alexa Fluor 568	Thermo Fisher Scientific	A10042	Immunofluorescence
Donkey anti-rabbit, Alexa Fluor 647	Thermo Fisher Scientific	A31573	Immunofluorescence
β-Actin	Cell Signaling Technology	3700 (ms), 8457 (rb)	Western blot
MSK1	Abcam	ab99412	Western blot
MSK2	Cell Signaling Technology	3679	Western blot, immunofluorescence
Phospho-MSK2 (Ser^360^)	Thermo Fisher Scientific	PA5-105431	Western blot
Phospho-ERK1/2 pathway antibody kit [phospho–c-Raf, phospho-MEK1/2, phospho-p90RSK, phospho-p44/42 MAPK(ERK1/2)]	Cell Signaling Technology	#9911T	Western blot
Anti-rabbit HRP	Cell Signaling Technology	7074S	Western blot secondary
Anti-mouse HRP	Cell Signaling Technology	7076S	Western blot secondary
