# Supplementary material for: Growth factor–induced activation of MSK2 leads to phosphorylation of H3K9me2S10 and corresponding changes in gene expression
Source: Sci Adv. 2024 Mar 13;10(11):eadm9518. doi: 10.1126/sciadv.adm9518 (PMC10936876; doi:10.1126/sciadv.adm9518)
Supplement: Supplementary file 1 — Figs. S1 and S2 Legends for data S1 and S2 [file sciadv.adm9518_sm.pdf]

Science Advances
AAAS

Supplementary Materials for

Growth factor–induced activation of MSK2 leads to phosphorylation of H3K9me2S10 and corresponding changes in gene expression

Karen G. Wong *et al.*

Corresponding author: Jonathan A. Epstein, epsteinj@pennmedicine.upenn.edu

Sci. Adv. **10**, eadm9518 (2024)
DOI: 10.1126/sciadv.adm9518

The PDF file includes:

Figs. S1 and S2
Legends for data S1 and S2

Other Supplementary Material for this manuscript includes the following:

Data S1 and S2

Supplemental Figure 1

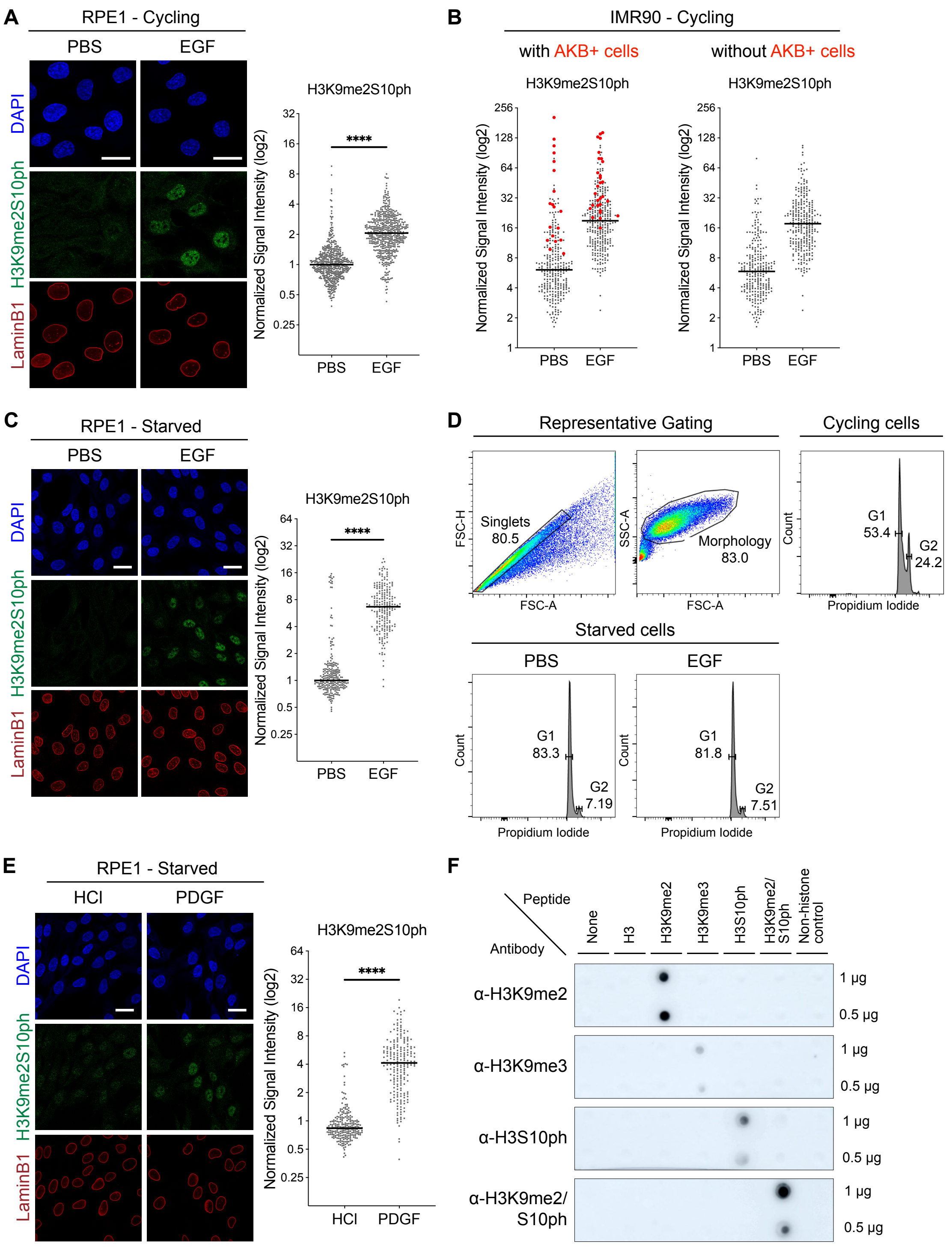

Fig. S1.
(A) Representative confocal images of cycling RPE1 cells immunostained for H3K9me2S10ph (green), Lamin B1 (red), and counterstained with DAPI (blue). Cells were treated with vehicle control (PBS) or EGF for 30 minutes. Dot plot shows distribution of H3K9me2S10ph signal

intensities per individual nucleus, normalized to the mean intensity of the vehicle control sample. Lines indicate median values. n≥528 cells per condition. Scale bars, 25μm.
(B) Dot plots show normalized H3K9me2S10ph signal intensities from cycling IMR90 cells immunostained for H3K9me2S10ph and Aurora B Kinase (AKB). Cells were treated with vehicle control (PBS) or EGF for 30 minutes. Distributions of H3K9me2S10ph including cells positive for AKB (red, left panel) or excluding AKB-positive cells (right panel). Lines show median values. n≥282 cells per condition.
(C) Representative confocal images of serum starved RPE1 cells immunostained for H3K9me2S10ph (green), Lamin B1 (red), and counterstained with DAPI (blue). Cells were treated with vehicle control (PBS) or EGF for 30 minutes. Dot plot shows distribution of H3K9me2S10ph signal intensities per individual nucleus, normalized to the mean intensity of the vehicle control sample. Lines indicate median values. n≥208 cells per condition. Scale bars, 25μm.
(D) Cell cycle distribution by flow cytometry using propidium iodide staining. IMR90 cells were treated with non-template control siRNA and either PBS or EGF for 30 minutes. Cycling cells were untreated.
(E) Representative confocal images of serum starved RPE1 cells immunostained for H3K9me2S10ph (green), Lamin B1 (red), and counterstained with DAPI (blue). Cells were treated with vehicle control (HCl) or PDGF for 30 minutes. Dot plot shows distribution of H3K9me2S10ph signal intensities per individual nucleus, normalized to the mean intensity of the vehicle control sample. Lines indicate median values. n≥214 cells per condition. Scale bars, 25μm.
(F) Specificity of antibodies was confirmed by immunoblot analysis. Each antibody shows specificity to detect the H3 tail peptide with relevant histone modification as indicated. See Methods for details.
Statistical analyses performed using unpaired t-test; **** p value < 0.0001

Supplemental Figure 2

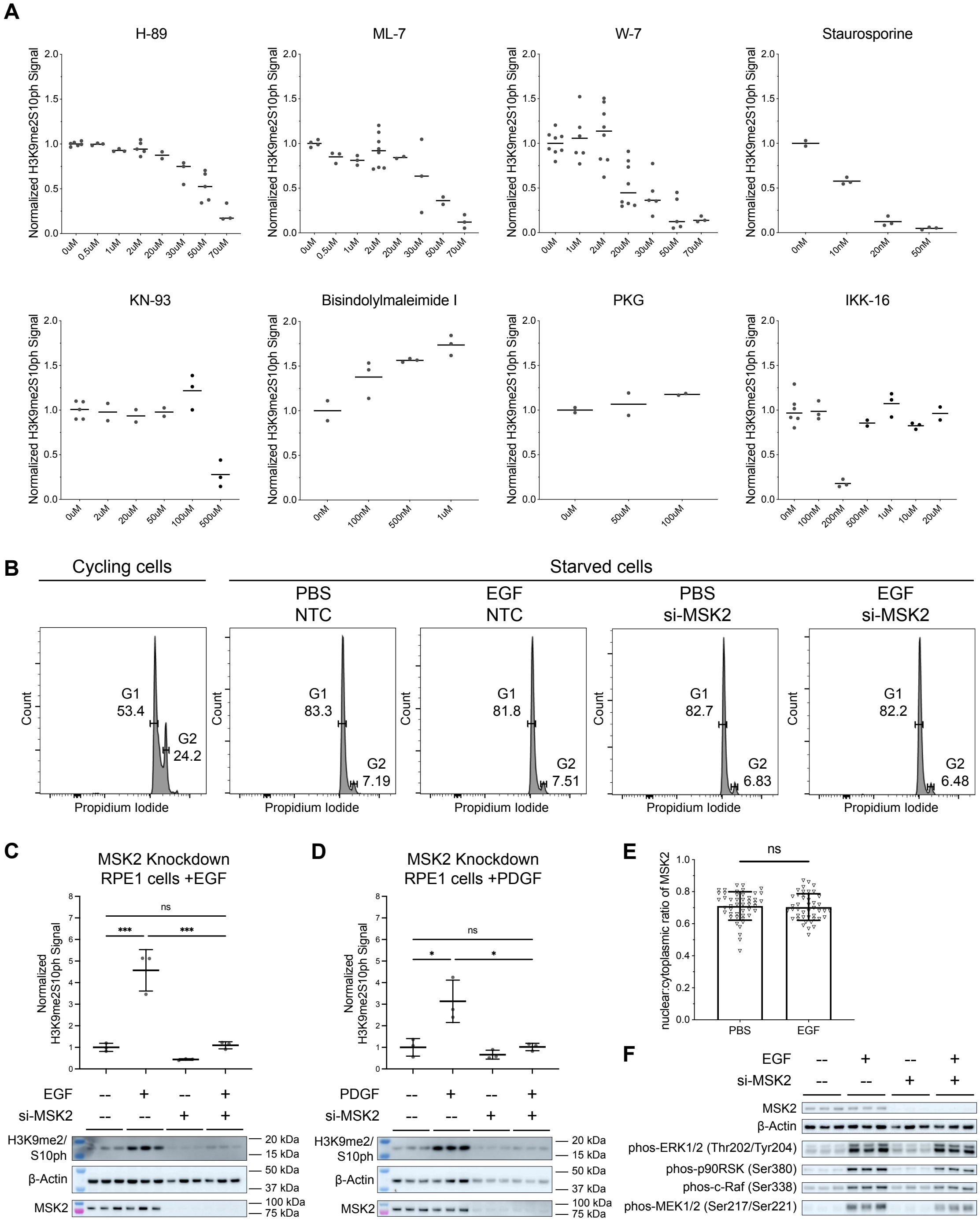

Fig. S2.

(A) Dot plot shows quantifications of H3K9me2S10ph Western blot signal from cycling IMR90 cells treated with the indicated chemical compounds at the indicated range of concentrations. Measurements were normalized to loading control (β-Actin or H3) and the mean of the untreated vehicle control sample. Lines indicate mean.

(B) Cell cycle distribution by flow cytometry using propidium iodide staining. IMR90 cells were treated with PBS or EGF and siRNAs targeting MSK2 as indicated. Panels showing cycling cells and cells treated with non-template controls (NTC) are duplicates from fig. S1C and included here for comparison purposes.
(C) Dot plot shows quantification of H3K9me2S10ph Western blot signal from serum starved RPE1 cells treated with EGF and siRNAs targeting MSK2 as indicated. Measurements were normalized to loading control (β-Actin) and the mean of the PBS sample. Lines indicate mean ± standard deviation. n=3 per condition. Representative Western blots shown below.
(D) Dot plot shows quantification of H3K9me2S10ph Western blot signal from serum starved RPE1 cells treated with PDGF and siRNAs targeting MSK2 as indicated. Measurements were normalized to loading control (β-Actin) and the mean of the PBS sample. Lines indicate mean ± standard deviation. n=3 per condition. Representative Western blots shown below.
(E) Quantification of the nuclear-to-cytoplasmic ratio of MSK2 immunofluorescence signal (as in Fig. 2F; see Methods) in cells treated with PBS or EGF for 30 minutes. Lines indicate mean ± standard deviation. n≥44 cells per condition.
(F) Western blots show activated forms of the Erk1/2 pathway members (Erk1/2, p90RSK, c-Raf, MEK1/2) from serum starved IMR90 cells treated with EGF and siRNAs targeting MSK2 as indicated. Statistical analyses performed using Two-Way ANOVA (panels C, D) or unpaired t-test (E); *** p value < 0.001, * p value < 0.05, ns: not significant.

Data S1. (separate file)
List of differentially expressed genes in serum starved IMR90 cells treated with EGF for 30 or 60 minutes.

Data S2. (separate file)
List of differentially upregulated genes in serum starved IMR90 cells treated with EGF for 60 minutes. "Gene cluster" column indicates which hierarchical cluster each gene belongs to as shown in Fig. 3E.
